# Evaluación de herramientas de implementación de la Guía de Práctica Clínica de infecciones de transmisión sexual

**DOI:** 10.26633/RPSP.2017.49

**Published:** 2017-04-20

**Authors:** Jaime Hernán Rodríguez Moreno, Antonio José Romero Vergara, Danilo De Jesús De Alba De Moya, Hernán Javier Jaramillo Rojas, Claudia Milena Díaz Rojas, Agustín Ciapponi

**Affiliations:** 1 Instituto de Evaluación Tecnológica en Salud La correspondencia se debe dirigir a: Jaime Hernán Rodríguez Moreno Bogotá Colombia Instituto de Evaluación Tecnológica en Salud, Bogotá, Colombia. La correspondencia se debe dirigir a: Jaime Hernán Rodríguez Moreno.; 2 Centro Cochrane - Instituto de Efectividad Clínica y Sanitaria Centro Cochrane - Instituto de Efectividad Clínica y Sanitaria Argentina Centro Cochrane - Instituto de Efectividad Clínica y Sanitaria, Argentina.

**Keywords:** Implementación de plan de salud, guía de práctica clínica, infecciones de transmisión sexual, Health plan implementation, practice guideline, sexually transmitted diseases

## Abstract

**Objetivo.:**

Establecer la aceptabilidad, percepción de utilidad y la implantación de las herramientas de implementación y el acompañamiento técnico del Instituto de Evaluación Tecnológica en Salud (IETS) en los hospitales de dos regiones de Colombia.

**Métodos.:**

Se desarrolló acompañamiento para la implementación de la Guía de Práctica Clínica (GPC) en 24 hospitales (17 de Antioquía y 7 de Cundinamarca) en zonas de alta prevalencia de infecciones de transmisión sexual (ITS), así como la instalación de las herramientas de implementación. Se aplicaron encuestas a los profesionales y entrevistas a los referentes.

**Resultados.:**

Del total de los encuestados, 86% conocen la GPC, 86% las recomendaciones trazadoras, 79% los flujogramas interactivos, 82% las hojas de evidencia y 41% nunca utilizó las herramientas de implementación. De los encuestados que han utilizado las herramientas, 55% lo hacen en el ordenador del sitio de trabajo, mientras que 24% utiliza su teléfono personal. Las herramientas de mayor utilidad son las hojas de evidencia y los flujogramas con 98% y las recomendaciones trazadoras con 92%. Las de más baja utilidad son las herramientas de impacto presupuestal (81%).

**Conclusiones.:**

Las herramientas de implementación y el acompañamiento técnico en los hospitales de dos regiones de Colombia se perciben como útiles y aceptables, aunque el grado de implantación es bajo. Los hallazgos de esta investigación contribuirán para que los diferentes actores, como el Ministerio de Salud y Protección Social, el IETS y el Departamento Administrativo de Ciencia, Tecnología e Innovación (Colciencias) entre otros, puedan mejorar los programas de implementación de guías de práctica clínica.

## Abstract (en)

***Objective*.:**

Determine the acceptability, perceived usefulness, and adoption of implementation tools and technical assistance provided by the Health Technology Assessment Institute (IETS) in hospitals in two regions of Colombia.

Assistance was provided for implementation of clinical practice guidelines (CPGs) in 24 hospitals (17 in Antioquia and seven in Cundinamarca) in areas with high prevalence of sexually transmitted infections, and for use of the implementation tools. Health professionals were given surveys and medical specialists were interviewed.

***Results*.:**

Overall, 86% of respondents are familiar with the GPGs, 86% with the tracer recommendations, 79% with the interactive flow charts, and 82% with the evidence sheets, while 41% never used the implementation tools. Of the respondents who used the tools, 55% did so on their work computer, while 24% used their personal telephone. The most useful tools were the evidence sheets and flow charts (98%) and the tracer recommendations (92%). The least useful were the budgetary impact tools (81%).

***Conclusions*.:**

The implementation tools and technical assistance provided in hospitals in two regions of Colombia are perceived as useful and acceptable, although the degree of implementation is low. The findings of this research will help the different actors, such as the Ministry of Health and Social Protection, the IETS, and the Administrative Department of Science, Technology and Innovation (Colciencias), among others, improve their programs for the implementation of clinical practice guidelines.

Las infecciones del tracto genital, entre las que se encuentran las infecciones de transmisión sexual (ITS), son una de las principales causas de consulta en la población adulta. Según la Organización Mundial de la Salud (OMS) (1), cada año ocurren 448 millones de casos nuevos de ITS en adultos entre 15 a 49 años en todo el mundo. Los principales microorganismos causantes son: *Treponena pallidum*, *Neisseria gonorroheae*, las clamidias y las tricomonas (no se incluye casos de infecciones por virus de inmunodeficiencia humana u otras ITS).

En Colombia, el informe sobre la situación de las ITS (1976–2000) considera que con una prevalencia de 6% de infección por *Chlamydia trachomatis*, se incurrió en costos superiores a 28 millones de dólares estadounidenses (USD) y, con una incidencia de 1,7% de infección por *N. gonorrhoeae*, los costos fueron cercanos a los USD 7 millones para el mismo período (2).

De acuerdo con lo indicado en el informe “Situación de las infecciones de transmisión sexual diferentes al VIH, Colombia, 2009–2011” publicado por el Ministerio de Salud de ese país, las infecciones de transmisión sexual constituyen un grave problema de salud pública. Mediante los registros de atención se reporta un promedio anual de 98 423 casos. Uno de los problemas más relevantes es la sífilis en gestantes, con una prevalencia de 1,7%, cifra mayor a la de 1% establecida por la Organización Panamericana de la Salud (OPS) como criterio de base (3).

La encuesta nacional de demografía y salud (4) mostró que, dentro de la población femenina entrevistada, 9% de las encuestadas habían presentado síntomas relacionados con ITS en los 12 meses anteriores a la realización de la encuesta.

El sistema de salud en Colombia está compuesto por entidades regulatorias como el Ministerio de Salud, entidades aseguradoras denominadas empresas promotoras de salud y prestadores de servicios de salud. En este esquema, corresponde a los hospitales la elaboración de guías y protocolos de manejo, así como su implementación, y a las aseguradoras, gobiernos locales y entidades del gobierno nacional supervisar su aplicación.

Desde el año 2007, el Ministerio de Salud y Protección Social y el Departamento Administrativo de Ciencia, Tecnología e Innovación (Colciencias) han invertido alrededor de 10 millones de dólares en el desarrollo de más de 50 guías de práctica clínica (GPC) con las metodologías más rigurosas. Una de las guías desarrolladas con financiamiento del Ministerio fue la GPC para el abordaje sindrómico del diagnóstico y tratamiento de los pacientes con infecciones de transmisión sexual y otras infecciones del tracto genital, publicada en el año 2013.

Con el objeto de fortalecer las competencias y la capacidad de implementación de las guías, el gobierno nacional ha desarrollado diversas estrategias, como la inclusión de las GPC como un componente obligatorio del sistema de calidad y actividades de diseminación pasiva en todo el país. Sin embargo, esta inversión no ha sido correspondida por un esfuerzo de las mismas dimensiones en el proceso de implementación, lo que puede llevar a que las guías sean “letra muerta” y que no redunden en un mejoramiento de la calidad de la atención en salud.

Para facilitar la implementación de las guías y mejorar la calidad de los servicios de salud, el IETS ha desarrollado herramientas como recomendaciones trazadoras (recomendaciones más importantes de la guías), hojas de evidencia (resumen de evidencia que justifica la puesta en práctica de las recomendaciones en lenguaje sencillo de fácil comprensión), flujogramas dinámicos, (flujogramas en línea que permiten interactuar fácilmente) indicadores y herramienta de análisis de impacto presupuestal (herramienta de planificación de la gestión administrativa y financiera) que ayudan a los integrantes del sistema a disminuir la brecha entre la evidencia y la práctica clínica.

El proyecto tiene por objeto mejorar la calidad de la atención en salud de las personas con infecciones de transmisión sexual a través de la implementación de la guía. El objetivo del estudio es establecer la aceptabilidad, percepción de utilidad y el grado de implantación de las herramientas de implementación y el acompañamiento técnico de IETS en los hospitales de dos regiones de Colombia.

## MÉTODOS

Este trabajo forma parte de una nueva iniciativa: “Mejoras en la ejecución de programas a través de investigaciones integradas en los mismos acerca de su ejecución (iPIER)”, desarrollado por la Alianza para la Investigación en Políticas y Sistemas de Salud (AHPSR), en colaboración con la OPS. El modelo iPIER jerarquiza a los ejecutores de programas como agentes clave de investigación con el objetivo de entender las fallas en los sistemas de salud que crean barreras a la implementación, así como permite identificar las soluciones a estas barreras. La investigación sobre la ejecución de programas integrada en los procesos existentes apoya su efectividad y políticas de salud eficaces a través de la utilización de la investigación que se llevó a cabo como parte del proceso de implementación. Una descripción detallada de la aplicación de la metodología de investigación se incluye en el documento conceptual iPIER (Evaluación de la aplicación de herramientas de implementación para promover la adopción de la Guía de Práctica Clínica [GPC] de infecciones de transmisión sexual en Antioquia y Cundinamarca, Colombia).

### Equipo de trabajo

El equipo de trabajo estuvo integrado por miembros del IETS: médico líder del proyecto con formación en proyectos sociales y administración en salud, dos médicos especialistas en implantación con formación en consultoría, un epidemiólogo clínico y un gerente de sistemas de información en salud. Además, iPIER proporcionó apoyo metodológico a través de personal del Instituto de Efectividad Clínica y Sanitaria (IECS).

### Componente ético

El protocolo de investigación fue aprobado por el comité de ética de investigación del Hospital Universitario de La Samaritana en Bogotá, Colombia.

### Diseño del estudio

**FIGURA 1.**
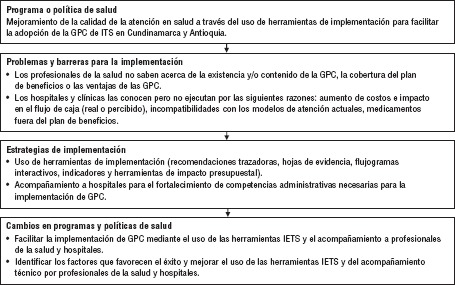
Diagrama de flujo del programa actual

Se trata de un estudio cualitativo descriptivo. El protocolo de investigación contempló el acompañamiento en implementación y el uso de herramientas de implementación para los hospitales incluidos (figuras 1 y 2): acompañamiento a hospitales para la implementación de GPC en la realización de talleres y acompañamiento para la identificación de barreras y facilitadores, construcción y puesta en práctica de los planes de implementación en cada institución. También acompañó mediante la instalación y puesta en funcionamiento de las herramientas de implementación del IETS (recomendaciones trazadoras, hojas de evidencia, flujogramas interactivos, indicadores y herramientas de impacto presupuestal) en cada hospital.

Las intervenciones fueron realizadas entre marzo y abril de 2015. En cada región, un especialista de implementación desarrolló las actividades por nodos: uno en Cundinamarca y cinco en Antioquia. La organización de estos nodos fue definida por integración geográfica.

Luego de la intervención se realizaron entrevistas a los líderes de proceso que participaron en los talleres y encuestas a los profesionales de la salud donde se instalaron las herramientas. Los datos cuantitativos fueron analizados mediante estadística descriptiva utilizando Google Survey®. Los datos cualitativos de las entrevistas se trascribieron, ordenaron y analizaron por tema (figura 3), y se identificaron los mensajes principales así como palabras y términos clave. La identificación se realizó con Microsoft Excel®.

Para el análisis de los datos se tuvieron en cuenta las siguientes condiciones:

***Fuente:*** elaboración propia, 2015.

GPC, guías de práctica clínica; IETS, Instituto de Evaluación Tecnológica en Salud; ITS; infecciones de transmisión sexual.

- Características de actores: identificación de las características de las instituciones, tales como el nivel de complejidad, los servicios involucrados, el número de sedes, la disponibilidad de ordenador en cada consultorio, la disponibilidad de internet y la especialidad de los profesionales.
- Características del sistema: se abordaron las relaciones existentes entre los hospitales y otros actores como los aseguradores y las entidades de vigilancia, y los factores de influencia de estos sobre la implementación de GPC.
- Evaluación de la percepción de utilidad y aceptabilidad a los referentes de los procesos que participaron en los talleres de implementación.

Los resultados se integrarán al proceso de implementación de GPC en todas sus fases:

- Fase de desarrollo de GPC: fortalecimiento del análisis de implementabilidad de las GPC (futuras y actualizaciones de las existentes).
- Fase de desarrollo de herramientas de implementación: información sobre la construcción y el ajuste de herramientas existentes o nuevas herramientas.
- Fase de implementación: mejora de la capacidad de los especialistas de implementación del IETS y facilitación de su interacción con las instituciones prestadoras de servicios de salud (IPS) y profesionales de la salud.

## RESULTADOS

El proyecto se inició con 24 instituciones (17 de Antioquia y siete de Cundinamarca) de las cuales 20 son hospitales de baja complejidad, tres de complejidad media y una de complejidad alta. Siete son de sede única y 17 tienen múltiples sedes (urbanas y rurales). La totalidad de las instituciones cuentan con médicos generales y profesionales de enfermería, ocho de ellas cuentan con especialistas en ginecología. Diecinueve de las 24 instituciones cuentan con ordenador en cada consultorio (solo en las sedes principales) y todas cuentan con acceso a internet, pero solo en áreas administrativas.

Los hospitales están organizados en redes de servicios (por proximidad geográfica) con un sistema de interacción denominado sistema de referencia y contrarreferencia: los hospitales de baja complejidad remiten a los hospitales de complejidad media y alta. Sin embargo, de acuerdo con las características del pagador (asegurador), esta interacción puede cambiar y tener niveles de referencia a otros hospitales públicos o privados de diferentes partes del departamento y no necesariamente a las involucradas en el proyecto.

A través de Google Docs® se enviaron 273 encuestas y se enviaron recordatorios semanales durante un mes. Se obtuvieron 74 respuestas (27%), tres respondientes manifestaron no participar en la encuesta. En el cuadro 1 se exponen los resultados.

Ochenta y seis por ciento conoce conocen la GPC, 86% las recomendaciones trazadoras, 79% los flujogramas interactivos y 82% las hojas de evidencia. Sin embargo, 41% de los encuestados nunca han utilizado las herramientas de implementación. En las barreras para aplicar las guías se encuentran elementos ajenos a los profesionales. Es de destacar que 50% de los hospitales no cuentan con los medicamentos y procedimientos, le siguen los tiempos de consulta programados no permiten la implementación (35%) y la forma de contratación de los hospitales (28%). Dentro de las acciones que favorecen la implementabilidad están la educación continua (92%), la educación interactiva (76%) y la auditoría (73%).

En relación con el uso, 55% de los usuarios manifiesta que ha usado las herramientas en el ordenador del sitio de trabajo, mientras que 24% utiliza su teléfono personal y 11% en otros tipos de dispositivos.

Las herramientas de mayor utilidad son las recomendaciones trazadoras y los flujogramas con (98%) y las hojas de evidencia (92%). Las de más baja utilidad son las herramientas de impacto presupuestal (81%). Con relación a la facilidad de uso, las dos terceras partes de los encuestados consideran que las herramientas son fáciles de utilizar, siempre que se reciba instrucción (cuadro 2).

Para evaluar la aceptabilidad y utilidad del acompañamiento, se recolectaron un total de 20 entrevistas. Cuatro instituciones no participaron por causas externas al proyecto, cómo cambios políticos, desinterés o falta de personal de las organizaciones para asumir los compromisos.

En relación con la utilidad, se indica que existe un alto grado de interés en el proceso de acompañamiento, con gran utilidad de las herramientas desarrolladas por el IETS, pero deja ver que las exigencias normativas hacen que la implementación, vista desde la perspectiva básica, incremente el trabajo de las organizaciones y se genere mayor necesidad de apoyo externo por parte de las entidades de asistencia técnica a nivel territorial.

**FIGURA 2.**
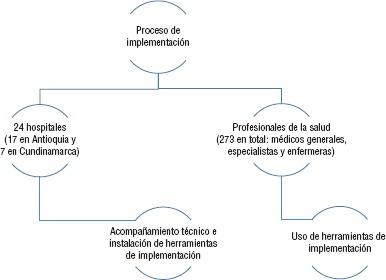
Esquema general del protocolo de investigación

***Fuente:*** elaboración propia 2015.

**FIGURA 3.**
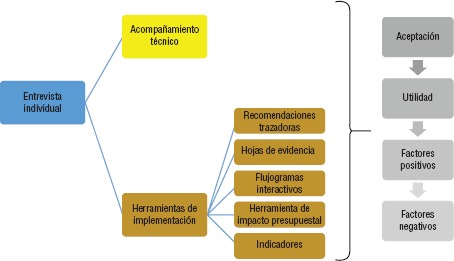
Plan de análisis de datos de las entrevistas

***Fuente:*** elaboración propia, 2015.

Entre los factores positivos más relevantes se encuentra que el IETS fortalece el trabajo de las organizaciones a través de estos acompañamientos. Las herramientas de implementación apoyan las actividades desarrolladas por los hospitales, pero se requiere mayor diseminación a estos desde los procesos formativos.

Entre los factores negativos se indica que para obtener un resultado positivo de estos procesos en los hospitales, se debe fortalecer la capacidad de trabajo de los profesionales dado que ellos tienen bajo nivel de compromiso. Los hospitales públicos tienen baja capacidad para poner en práctica procesos de implementación estructurados, por la baja disponibilidad de recursos, la alta rotación del talento humano y la voluntad política de los dirigentes.

Teniendo en cuenta los resultados, se establecieron los siguientes elementos en el programa de implementación de GPC:

- Inclusión del análisis del contexto político administrativo en las fases de implementabilidad y en el acompañamiento.
- Facilitar instrucciones a los profesionales para mejorar la comprensión de las herramientas de implementación.
- Lograr mayor interacción entre las diferentes herramientas de implementación para que sean más dinámicas.
- Desarrollo de herramientas de implementación adecuadas para dispositivos móviles tipo tableta y celulares inteligentes.
- Inclusión de las herramientas y las GPC en la formación del talento humano en salud.

## DISCUSIÓN

La implementación de guías de práctica clínica es una actividad obligatoria desde el punto de vista normativo en Colombia, pero novedosa en el alcance y la extensión dada por las normas que regulan el sistema de salud colombiano (5).

Para facilitar el proceso de implementación y acortar la brecha entre la teoría y la práctica, el ministerio desarrolló un manual para la implementación de guías de práctica clínica (6), publicado en el mes de marzo de 2014.

El proceso de implementación de evidencia debe ser activo, planeado, estratégico y con la participación de los interesados, entre los que se incluyen a los profesionales, las organizaciones prestadoras de servicios de salud y los tomadores de decisiones. Se debe valorar siempre la percepción y sus características de ellos para evaluar y mejorar la estructura de las actividades (7).

La definición de los criterios de evaluación se centraron en las categorías de corto plazo establecidas por Proctor y colaboradores (8), donde se indica que se puede mediar la usabilidad, aceptabilidad, facilidad de uso de las herramientas de implementación de manera temprana.

 A través de la caracterización y mapeo de los actores y del sistema se puede establecer que, si bien la implementación de las GPC es un elemento obligatorio, los hospitales presentan diversos elementos divergentes que generan barreras para que las actividades se hagan de forma adecuada. Entre las situaciones que generan problemas para la implementación se encuentran la rotación del personal asistencial, los problemas en el flujo de recursos económicos en los hospitales, el bajo nivel de conocimiento acerca de los procesos de implementación y de forma muy importante la dualidad de directrices que existen entre las entidades rectoras del sistema, las instituciones que vigilan y las organizaciones que contratan y pagan por los servicios prestados. Este elemento se correlaciona con los hallazgos de McKillop, donde indica que la implementación es contextual y que esta depende del trabajo del equipo de implementación (9). El uso de herramientas de implementación es un elemento que facilita el proceso en forma general, tal como lo describe Yang en el estudio de la implementación de guías para la enfermedad tiroidea (10).

Como se observa en las entrevistas dirigidas a los referentes de las instituciones y en los aspectos obtenidos en las encuestas, el trabajo y desarrollo de las herramientas del IETS, facilitan la comprensión del proceso, pero la baja disponibilidad de recursos lleva a que las organizaciones no garanticen los insumos necesarios para aplicar las recomendaciones de las GPC. Este hecho es coincidente con el PARish Framework, donde se indica que uno de los elementos centrales para el proceso de implementación es el contexto de la entidad implementadora (11).

Por último, Bohmer (12) indica tres situaciones a tener en cuenta en relación con el bajo éxito de la implementación en las organizaciones: la primera hace referencia a no saber qué hacer, la segunda a no hacer lo que sabemos y la tercera indica que incluso cuando sabemos que hacer y hacemos lo que sabemos, no lo hacemos bien. Estas situaciones se relacionan con las consideraciones expresadas por los profesionales donde indican que, al aumentar los procesos de educación continuada e interactiva, se favorecen los procesos de implementación, este hecho contradictorio con los resultados de la encuesta, donde consideran que las principales barreras no son de conocimiento sino de gestión organizacional.

### Fortalezas y debilidades

Dentro de las características del proyecto se observa que el acompañamiento a las instituciones es adecuado, pero su capacidad para recibirlo no es óptimo. Esto se observa en el hecho que los referentes claves participaron de manera extensa en el proceso y en la respuesta a la entrevista, mientras que los profesionales de la salud que participaron, mostraron bajo interés en la respuesta a la encuesta (27%), lo cual puede generar baja capacidad para generalizar las respuestas obtenidas.

El desarrollo de una estructura normativa que obliga a los prestadores de salud a mejorar la implementación de las GPC permite que el desarrollo de actividades de acompañamiento y las herramientas, con los cambios generados permitan fortalecer los resultados finales en el paciente. Este último elemento permite que este tipo de actividades perduren en el tiempo y sean incluidos en otras políticas y normativas del sistema de salud.

## CONCLUSIONES

Las herramientas de implementación y el acompañamiento técnico se perciben como útiles y aceptables, pero el grado de implantación es bajo en los hospitales del proyecto. Los hallazgos contribuirán para que el Ministerio de Salud y Protección Social, el IETS y Colciencias, entre otros, puedan incrementar la generación de estas herramientas; a los hospitales para que en sus programas de implementación de GPC desarrollen estrategias activas de transferencia de información y para que los demás tomadores de decisiones estén en capacidad de generar mayores acciones para la implementación y cambios en la práctica clínica de los participantes.

## Agradecimientos

La Organización Panamericana de la Salud brindó cooperación técnica para el desarrollo de este proyecto. En el contexto del programa iPIER, el Instituto de Efectividad Clínica y Sanitaria (IECS) brindó asistencia técnica para el desarrollo del protocolo, la ejecución y publicación del proyecto.

## Financiamiento

Este trabajo fue financiado por la Alianza para la Investigación en Políticas y Sistemas de Salud (AHPSR), de la Organización Mundial de la Salud (OMS) y la Organización Panamericana de la Salud (OPS).

## Declaración

Las opiniones expresadas aquí son responsabilidad de los autores y no reflejan necesariamente el criterio ni la política de la Organización Panamericana de la Salud/Organización Mundial de la Salud.
